# Predicting red blood cell transfusion for primary hip and knee arthroplasty: Designing a hub and spoke high-volume arthroplasty model

**DOI:** 10.1016/j.jcot.2025.102974

**Published:** 2025-03-12

**Authors:** Edward Hayter, Vijay Badial, Reece Barter, Harry Hodgson, Raymond E. Anakwe

**Affiliations:** a Department of Trauma and Orthopaedic Surgery, St Mary's Hospital, Imperial College Healthcare NHS Trust, Praed Street, London, W2 1NY, UK; b Imperial College, London, UK

**Keywords:** Arthroplasty, Transfusion, Patient pathway, Hip replacement, Knee replacement

## Abstract

**Background:**

Waiting lists for elective surgery are at record levels. Consolidating elective care into superhubs where high-volume surgery can be undertaken efficiently is an attractive solution. These pathways should be safe but also efficient and convenient for patients.

We undertook this study to develop a model that would reliably predict healthy (American Society of Anaesthesiologists [ASA] class 1 and 2) patients who could be treated on a streamlined pathway and who would reliably not require red blood cell transfusion during their inpatient admission.

**Methods:**

We retrospectively identified all patients undergoing primary total hip arthroplasty (THA) or total knee arthroplasty (TKA) at our centre over a five-year period. We used binary logistic regression to develop a predictive model based on these variables.

**Results:**

We identified 13-preoperative candidate variables from our literature search and used these to construct our predictive model. The final validated model was highly effective in predicting those patients who would not need a red blood cell transfusion (area under curve = 0.945).

**Conclusion:**

Our model reliably predicts those healthy (ASA 1 or 2) patients undergoing primary THA or TKA surgery who will not require a red blood cell transfusion during their admission. These patients are suitable for this streamlined pathway without the need for unnecessary preoperative patient tests and travel.

**Level of evidence:**

III.

## 1 Introduction

Waiting lists for planned elective surgery in the United Kingdom (UK) are the largest they have been since 2008, with seven million patients facing extended waits for planned elective surgery.^1^ The National Health Service (NHS) has not met its target of 92 % of patients waiting a maximum of 18 weeks, from referral to treatment, since 2015.^1^ Those waiting for an elective orthopaedic operation are on the largest waiting list of any surgical specialty in the UK and those waiting for primary total hip arthroplasty (THA) and primary total knee arthroplasty (TKA) are on the longest list.^2^^,^^3^ THA and TKA numbers are predicted to increase in the United Kingdom by 37.7 % and 36.6 %, respectively, from 2018 levels to 2060 according to data from the National Joint Registry.^4^^,^^5^

A key strategy to reduce waiting lists and prolonged waiting times is the centralisation of planned elective surgery for healthy adults (American Society of Anaesthesiologists [ASA] grades 1 and 2) to be undertaken in regional elective surgical hubs.^6^^,^^7^ These surgical hubs are designed to be high volume, low complexity centres, devoted to planned surgery and protected from the pressures of acute unplanned and emergency care.^8^ Consolidating these services into regional hubs allows for the concentration of experience in surgical, operative and rehabilitation teams, the development of efficient and streamlined pathways and for extended patient waiting times to be addressed in the most efficient and equitable way possible.

The patient pathway begins in local and community hospitals and services. All pre and post operative outpatient appointments, tests and rehabilitation are undertaken locally. The patient only attends the surgical hub on the day of surgery where surgery is undertaken by their local surgical team. This maintains local patient focussed care, avoids unnecessary travel for the patient and improves equity and access for patients who may find it difficult to undertake repeated travel.

Modern surgical and anaesthetic practice has been developed to reduce the transfusion requirements associated with primary joint arthroplasty surgery. Being able to reliably predict those patients who will not need a perioperative transfusion of blood products could improve the efficiency and safety of theatre scheduling.

We undertook this study to develop a reliable predictive model to identify patients graded ASA 1 and 2 who reliably would not require a perioperative red blood cell transfusion while undergoing THA or TKA.

## 2 Materials and methods

### 2.1 Study population

We retrospectively identified all patients who underwent primary THA or TKA at our institution over a five-year period between January 2018 and February 2023. We reviewed the patient record of care for each patient. Five-hundred and thirty-four patients underwent surgery over this period of whom 315 patients underwent THA. We excluded two patients who were undergoing revision or secondary procedures and 142 patients who were graded as ASA grade 3 or 4. This left 417 patients who were included to form the training data set for our model. In undertaking this study we adopted the Transparent reporting of a multivariable prediction model for individual prognosis or diagnosis (TRIPOD) standards and statement for reporting.^9^ The study was approved by our institutional review board. Patient participants provided informed consent.

The model was validated using an independent data set of ASA 1 and 2 patients undergoing primary THA or TKA at our institution. 136 patients underwent surgery between March and May 2023. One patient undergoing a secondary procedure was excluded as were 34 patients who were graded as ASA 3 or 4. This left a group of 101 patients who formed our validation set. There were no differences in study setting, eligibility criteria, outcome measure and predictors between the training and validation sets.

### 2.2 Data collection and variable selection

Our outcome measure was defined as red blood cell transfusion either intraoperatively or postoperatively until hospital discharge. We undertook a English language literature review using Medline (Pubmed), EMBASE and the Cochrane Central trials register to identify published studies and trials which evaluated factors predictive of blood product transfusion in patients undergoing primary THA or TKA. From our literature review, we identified 13 candidate variables available pre-operatively: seven categorical and six continuous (Table 1). Data was extracted from the patient record of care; no data was missing.

**Table 1:** Comparison of demographics, candidate variables and outcome measure between the training and validation data sets.

		Training data set (n = 417)	Validation data set (n = 101)	p-value
Candidate variables	Gender (n) – Male/Female	161/256	38/63	0.909^a^
	Age (yrs) – mean (SD)	68 (10)	73 (8)	<0.001^b^
	ASA grade – I/II	390/27	91/10	0.279^a^
	Cardiac medical history including hypertension in isolation - n (%)	104 (25 %)	23 (23 %)	0.700^a^
	Cardiac medical history excluding hypertension in isolation - n (%)	32 (8 %)	14 (14 %)	0.077^a^
	Anticoagulation use – n (%)	60 (14 %)	3 (3 %)	<0.001^a^
	BMI (kg/m^2^) – mean (SD)	29 (10)	29 (6)	0.027^b^
	Height (m) – mean (SD)	1.6 (0.2)	1.7 (0.2)	0.001^b^
	Weight (kg) – mean (SD)	81 (15)	83 (16)	0.168^b^
	Haemoglobin (g/L) – mean (SD)	134 (15)	121 (12)	<.001^b^
	Platelet count (10^9^/L) – mean (SD)	260 (71)	252 (56)	0.262^b^
	Surgical site – TKA/THA	186/231	46/55	0.911^a^
	Cemented prosthesis – n (%)	276 (66 %)	71 (70 %)	0.480^b^
	RBC transfusion – n (%)	53 (13 %)	11 (11 %)	0.737^b^

Values are expressed as means. Statistical significance is calculated using Fischer's exact test for binary metrics and using the unpaired *t*-test for continuous data.

a Fischer's exact.

b Unpaired *t*-test.

### 2.3 Statistical analysis

A binary logistic regression model was created using the training data set, including all candidate variables. The classification cutoff value was set at 0.9. Backwards stepwise elimination of variables, in order of least significance, was used to select variables to include in the final predictive model. Variables were eliminated if their exclusion resulted in a statistically insignificant drop in the log likelihood ratio (p > 0.05). The process terminated, and the excluded variable was not eliminated, when the drop in loglikelihood ratio was significant (p ≤ 0.05). Wald Chi-Squared Test using a 2-tailed p-value was used to evaluate significance of included variables individually. Model calibration was evaluated by the Nagelkerke R Square value and the Hosmer and Lemeshow Test. Model performance was assessed by measuring the Area Under the Curve (AUC) of the Receiver Operating Characteristic (ROC) curve with 95 % confidence intervals.

Significant difference between the training and validation data sets was evaluated for each candidate variable: unpaired *t*-test was used for continuous variables; Fischer's exact test was used for binary variables. The predictive model created using the training data set underwent temporal validation using the independent validation data set. Discrimination of the model was evaluated by calculating the AUC of the ROC curve. Data analysis was performed using SPSS 29.0 software (SPSS Inc, Chicago, Ill).

## 3 Results

417 patients were included in the study group used to develop the predictive model. Of these, 256 (61 %) were women. 231 (55 %) patients underwent primary THA and the rest underwent primary TKA. Patient demographics as well as the mean values for the 13 candidate pre-operative variables included in the predictive model are described in Table 1.

Stepwise regression analysis reduced the number of pre-operative predictive variables from 13 to six until further analysis no longer improved the model. The variables that remained in the model were ASA status, previous cardiac medical history (excluding hypertension in isolation), weight, height, preoperative haemoglobin level and preoperative platelet level. All variables selected by backwards stepwise elimination were included in the final predictive model irrespective of individual p-value to optimise performance of the model and to account for interactions between individual variables.^10^ The fit of the model remained good with reduction from 13 to six variables (Nagelkerke R Square value = 0.725; Hosmer and Lemeshow Test = 0.960) (Table 2).

**Table 2:** Characteristics of predictive model.

Included preoperative variables	β, regression coefficient	p-value^a^
ASA 2	−2.346	0.076
Cardiac medical history present (excluding hypertension in isolation)	2.562	0.001
Height (m)	−10.88	<0.001
Weight (kg)	0.05707	0.005
Haemoglobin (g/L)	0.1912	<0.001
Platelet count (109/L)	−0.005451	0.068
Model intercept (constant)	−5.966	0.109

Statistical significance tested using the Wald Chi-Squared with a 2-tailed p-value to 3 decimal places.

a Wald Chi-Squared test.

**Fig. 1:**
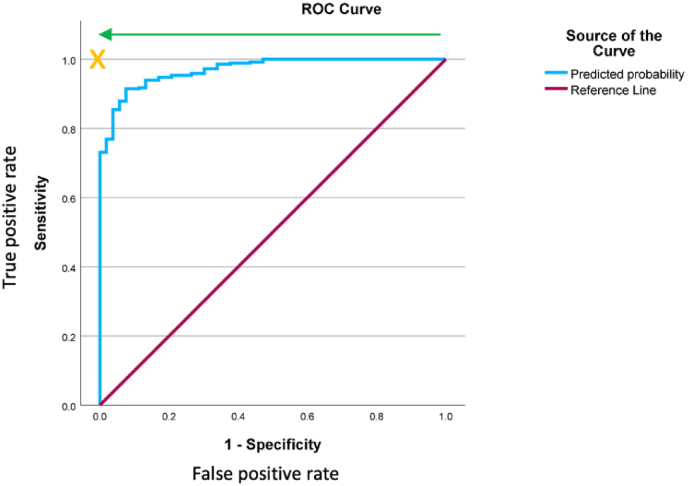
ROC Curve for Predictive model (Training data set) AUC = 0.970.

The model predicted that 329 of 417 (79 %) patients would not need a red blood cell transfusion. In fact, 325 patients of these patients did not require a transfusion. The positive predictive value (PPV) for the model is 99 %**.** The negative predictive value for the model is 56 %. The model was highly effective at predicting those patients who would not require a red blood cell transfusion in the perioperative phase, AUC = 0.970 [95 % CI 0.954–0.987] (Fig. 1).

**Fig. 2:**
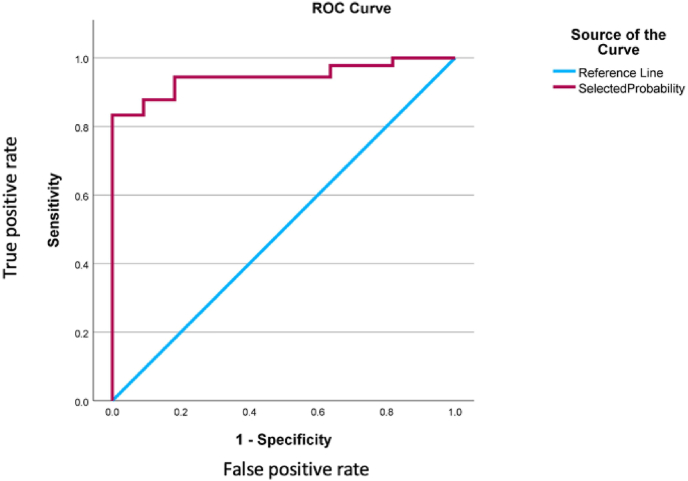
ROC Curve for Predictive model (validation data set) AUC = 0.950.

Testing and validation of the model with an independent data set confirmed that the model remained effective. The model predicted that 47 of 101 patients would not require a blood transfusion and in fact, none of these 47 patients were transfused. The PPV of the model is 100 %**.** Testing and validation of the model with an independent data set confirmed that it is highly effective at predicting those patients who would not require a blood transfusion in the perioperative phase, AUC = 0.945 [95 % CI 0.900–0.990] **(**Fig. 2). The negative predictive value of the model for the validation data set was 20 % (95 % CI 11–32 %).

## 4 Discussion

This study aimed to devise a predictive red blood cell transfusion model for ASA grade 1 and 2 patients undergoing primary THA or TKA, predicting with a high degree of accuracy those patients who would not require a transfusion in the perioperative period. The benefits of a practical and reliable model based on easily identified pre-operative factors are clear. The financial benefit of reducing unnecessary testing and group and save sampling for patients is attractive. In addition, a reliable and effective model will facilitate an efficient patient pathway which remains patient focussed and avoids unnecessary patient inconvenience and travel. There is good evidence that travel distance and time are key determinants for patient experience and acceptance of new models of care.^11^^,^^12^

Patients can be screened during list planning, using metrics and assessments obtained in the local pre-operative assessment to inform the predictive model via a smart phone or electronic health record interface. Those who are predicted to not require a blood transfusion can be safely placed first or early on the surgical operating list allowing the operating theatre to function without delays. Patients who are likely to require blood transfusion are scheduled later in the day to allow for on-the day group and save sampling.

In patient and stakeholder consultations around the movement of the service to a model where surgery is delivered at regional super-hubs, the principal concerns raised by patients were about travel links, transport time and the requirement for additional travel. There is evidence that increasing travel costs and time risks excluding sections of patients who are unable to access or afford increased travel costs and reluctant to accept treatment outside their local areas and support structure.^13^^,^^14^ This has been corroborated in local surveys, consultations and feedback from stakeholders.

Several studies have examined the potential for various patient and procedure-based variables to predict post-operative anaemia and the need for a blood transfusion after THA and TKA surgery.^15^^,^^16^ There are several advantages to the approach that we have taken in this study. The use of a multivariable model over a single predictive variable allows for an improved PPV while stepwise regression analysis to remove weak predictors simplifies the model and reduces the data inputs that are required while maintaining the effectiveness and reliability of the model. This is in line with the statistical principle of parsimony. Interestingly age, sex and BMI were amongst the demographic candidate variables previously reported to inform similar predictive models however were found to be uninformative in our model. It is possible that the impact of these factors is relatively reduced for healthy (ASA 1 and 2) patients. The model is based on pre-operative factors that can be assessed in advance of surgery. Factors such as operating time, the use of surgical drains, tranexamic acid were not included.

Our model predicts the likelihood of not needing a transfusion. With a probability cut-off threshold of 0.90, the PPV and AUC for our model displays a high degree of accuracy in this prediction. This compares very favourably with other models predicting the likelihood of transfusion.^15^^,^^16^ By increasing the probability cut-off threshold, one is able to make the criteria ‘stricter’ when classifying patients as ‘will not need’ a transfusion resulting in fewer false positives. A higher predictive cut off results in more stringent selection however a lower number of predictions rending the model less useful. A balance between false positive rate and model utility was found. Our negative predictive value was poor, 56 % and 20 % for the training and validation sets respectively. Fortunately, the importance of this is small as our model has not been designed to predict those patients who will require a transfusion, but to reliably identify those who will not. Huang et al. reported a similar finding.^15^

Despite the high PPV there is a small false positive risk (i.e. a patient requiring a transfusion despite being predicted not to). This may lead to a delay to cross matched transfusion once a need is identified, however given the PPV of 99 % for the training set and 100 % for the validation set this would be a rare occurrence. Post operative transfusions tend to be administered in an urgent but not emergent manner allowing time for cross matching to be undertaken after a need is identified, and readily available universal blood can cover a true emergency.

There are a number of limitations to our study. The size of the training and validation data sets used to develop and validate our model were smaller than some others.^15^^,^^16^ Nevertheless, our model development and validation has produced a highly effective predictive model with better characteristics and reliability than previous attempts. A consequence of analysing recent data was that our data period spanned the period of the Coronavirus pandemic, when primary THA and TKA numbers were lower than usual. Our study is a single centre study and includes only ASA 1 & 2 patients, which is a practical limitation as this is the patient group intended for treatment using this hub and spoke model.

Our validation set was temporal, which as a process measure lies between true internal and external validation.^17^ We analyzed a separate cohort of patients who underwent primary THA and TKA from a more recent time period to those used in the training data set. We recognise that this validates our approach and our results as reproducible but provides only limited information on generalisability particularly in application to other populations and healthcare settings. To inform on broader generalisability, we would recommend further local or multicentre validation.^18^

Allogenic blood transfusion is not entirely benign. There is a risk of transfusion reaction, incorrect patient identification, the administration of incompatible blood products, circulatory overload and a relationship between blood transfusion and prosthetic joint infection.^19^^,^^20^ Generally, the risk of allogenic blood transfusion after primary joint arthroplasty is low, particularly for fit and healthy adult patients without significant comorbidities. Predictive modelling forms only part of an effective blood management strategy. Other strategies include patient haemoglobin pre-optimisation with oral iron supplementation, intravenous iron infusion, or erythropoietin stimulating agents; autologous blood banking; and intraoperative practices such as tourniquet use, cell salvage, controlled hypotension and routine tranexamic acid use. A comprehensive approach is required but our study shows that predictive modelling can be used effectively in this way and to support new models of care.

## 5 Conclusion

Our model demonstrates that it is possible to predict with a high degree of certainty those patients who will not require a red blood cell transfusion. This will be important to maintain the efficiency of a new regional model of care and also a patient-centred focus, minimising travel, travel time and unnecessary testing, improving the acceptability and accessibility of care. We believe that this could find application in a variety of global settings especially where surgical services are required to cover large geographical or remote areas.

## CRediT authorship contribution statement

**Edward Hayter:** Methodology, Validation, Formal analysis, Investigation, Data curation, Writing – original draft, Writing – review & editing. **Vijay Badial:** Methodology, Validation, Investigation, Writing – original draft, Writing – review & editing. **Reece Barter:** Methodology, Validation, Investigation, Writing – original draft, Writing – review & editing. **Harry Hodgson:** Methodology, Writing – original draft, Writing – review & editing. **Raymond E. Anakwe:** Conceptualization, Methodology, Validation, Formal analysis, Investigation, Data curation, Writing – original draft, Writing – review & editing.

## Consent to participate

Informed consent was obtained from all individual participants included in the study.

## Consent to publish

The authors affirm that human research participants provided informed consent for inclusion in this study.

## Availability of data and materials

Data is provided within the manuscript. Complete datasets used and/or analyzed are available from the corresponding author upon reasonable request.

## JCOT consent statement

The study was approved by our institutional review board. Patient participants provided informed consent.

## Ethical clearance

This study was performed in line with the principles of the Declaration of Helsinki. This is an observational study. The Clinical Audit and Effectiveness Committee has confirmed that no ethical approval is required.

## Source of funding

This study received no funding, and the authors have no financial conflict of interests to declare.

## Declaration of competing interest

The authors have no relevant financial or non-financial interests to disclose.

## References

[1] The King’s Fund Waiting times for elective (non-urgent) treatment: referral to treatment (RTT). https://www.kingsfund.org.uk/projects/nhs-in-a-nutshell/waiting-times-non-urgent-treatment#footnote1_t9i7iyr.

[2] British Orthopaedic Association A message from the British Orthopaedic Association to people waiting for joint replacement and other orthopaedic surgery. https://www.boa.ac.uk/resource/a-message-for-people-waiting-for-joint-replacement-and-other-orthopaedic-surgery.html.

[3] Royal College of Surgeons of England More than 2 million people waiting longer than statutory 18 weeks for NHS treatment. https://www.rcseng.ac.uk/news-and-events/media-centre/press-releases/waiting-times-press-notice-november-2021/.

[4] Matharu G., Culliford D., Blom A., Judge A. (2022). Projections for primary hip and knee replacement surgery up to the year 2060: an analysis based on data from the National Joint Registry for England, Wales, Northern Ireland and the Isle of Man. Ann R Coll Surg Engl.

[5] National joint Registry 16th annual report. https://reports.njrcentre.org.uk/Portals/0/PDFdownloads/NJR%2016th%20Annual%20Report%202019.pdf.

[6] NHS England (2022). Delivery plan for tackling the COVID-19 backlog of elective care. https://www.england.nhs.uk/coronavirus/wp-content/uploads/sites/52/2022/02/C1466-delivery-plan-for-tackling-the-covid-19-backlog-of-elective-care.pdf.

[7] Saklad M. (1941). Grading of patients for surgical procedures. Anesthesiology.

[8] NHS England Surgical hubs. https://gettingitrightfirsttime.co.uk/hvlc/surgical-hubs/.

[9] Collins G.S., Reitsma J.B., Altman D.G., Moons K.G.M. (2015). Transparent reporting of a multivariable prediction model for individual prognosis or diagnosis (tripod). Circulation.

[10] Shipe M.E., Deppen S.A., Farjah F., Grogan E.L. (2019). Developing prediction models for clinical use using logistic regression: an overview. J Thorac Dis.

[11] Ambroggi M., Biasini C., Del Giovane C., Fornari F., Cavanna L. (2015). Distance as a barrier to cancer diagnosis and treatment: review of the literature. Oncologist.

[12] Gupta S., Rastogi K., Bhatnagar A.R., Singh D., Gupta K., Choudhary A.S. (2018). Compliance to radiotherapy: a tertiary care center experience. Indian J Cancer.

[13] Henneman P.L., Garb J.L., Capraro G.A., Li H., Smithline H.A., Wait R.B. (2011). Geography and travel distance impact emergency department visits. J Emerg Med.

[14] Brundisini F., Giacomini M., DeJean D., Vanstone M., Winsor S., Smith A. (2013). Chronic disease patients' experiences with accessing health care in rural and remote areas: a systematic review and qualitative meta-synthesis. Ont Health Technol Assess Ser.

[15] Huang Z., Huang C., Xie J. (2018). Analysis of a large data set to identify predictors of blood transfusion in primary total hip and knee arthroplasty. Transfusion.

[16] To J., Sinha R., Kim S.W. (2017). Predicting perioperative transfusion in elective hip and knee arthroplasty: a validated predictive model. Anesthesiology.

[17] Ramspek C.L., Jager K.J., Dekker F.W., Zoccali C., van Diepen M. (2020). External validation of prognostic models: what, why, how, when and where?. Clin Kidney J.

[18] Meara J.G., Leather A.J., Hagander L. (2015). Global Surgery 2030: evidence and solutions for achieving health, welfare, and economic development. Lancet.

[19] Zhu Y., Zhang F., Chen W., Liu S., Zhang Q., Zhang Y. (2015). Risk factors for periprosthetic joint infection after total joint arthroplasty: a systematic review and meta-analysis. J Hosp Infect.

[20] Blanco J.F., Díaz A., Melchor F.R., da Casa C., Pescador D. (2019). Risk factors for periprosthetic joint infection after total knee arthroplasty. Arch Orthop Trauma Surg.

